# Prospective Intention-Based Lifestyle Contracts: mHealth Technology and Responsibility in Healthcare

**DOI:** 10.1007/s10728-020-00424-8

**Published:** 2021-01-11

**Authors:** Emily Feng-Gu, Jim Everett, Rebecca C. H. Brown, Hannah Maslen, Justin Oakley, Julian Savulescu

**Affiliations:** 1 Nursing and Health Sciences, Faculty of Medicine, Monash University, Melbourne, Australia; 2 Uehiro Centre for Practical Ethics, University of Oxford, Oxford, UK; 3 Monash Bioethics Centre, Monash University, Melbourne, Australia

**Keywords:** Responsibility, Healthcare, mHealth, Lifestyle contract, Resource allocation

## Abstract

As the rising costs of lifestyle-related diseases place increasing strain on public healthcare systems, the individual’s role in disease may be proposed as a healthcare rationing criterion. Literature thus far has largely focused on retrospective responsibility in healthcare. The concept of prospective responsibility, in the form of a lifestyle contract, warrants further investigation. The responsibilisation in healthcare debate also needs to take into account innovative developments in mobile health technology, such as wearable biometric devices and mobile apps, which may change how we hold others accountable for their lifestyles. Little is known about public attitudes towards lifestyle contracts and the use of mobile health technology to hold people responsible in the context of healthcare. This paper has two components. Firstly, it details empirical findings from a survey of 81 members of the United Kingdom general public on public attitudes towards individual responsibility and rationing healthcare, prospective and retrospective responsibility, and the acceptability of lifestyle contracts in the context of mobile health technology. Secondly, we draw on the empirical findings and propose a model of prospective intention-based lifestyle contracts, which is both more aligned with public intuitions and less ethically objectionable than more traditional, retrospective models of responsibility in healthcare.

**Supporting Information:**

The online version of this article (10.1007/s10728-020-00424-8) contains supplementary material, which is available to authorized users.

## Introduction

Healthcare expenditure has grown substantially in recent years [55], in part due to the rise of so-called ‘lifestyle diseases’ which are closely tied to behaviours such as tobacco use, harmful alcohol use, physical inactivity, and poor diet.

Individual responsibility for health has been proposed as a solution to unsustainable growth in public healthcare costs, with the language of individual responsibility becoming more prominent within health policies and guidelines across the developed world [16, 28, 43, 52]. The first examples of lifestyle-dependent healthcare access are beginning to appear [43], with access to treatments, including elective surgery or fertility treatment, being restricted based on patient factors such as body mass index (BMI) or smoking status [31, 50]. In another example, the (since discontinued) 2006 West Virginian Medicaid Reform saw insurance coverage being held conditional upon on the fulfilment of patient responsibilities [9]. While cost-effectiveness is often lauded as a justification for such policies, in the absence of evidence that such restrictions are truly based on a robust cost-effectiveness analysis [43, 50], it seems that underlying attitudes towards desert and moral responsibility for ill health may be playing a covert role in health policy.

As Sharkey and Gillam [46] have noted, progress on the responsibility in healthcare debate has stalled. Much of the bioethical literature focuses on whether people should be given lower priority access to healthcare if they are responsible for their disease. This is a *retrospective* model of responsibility; the focus is on past behaviours and whether they causally contributed to a disease in a way that makes that individual morally responsible. Feiring [20] proposes a different model based on the concept of *prospective* responsibility, which is akin to having a duty or obligation. In a prospective framework of responsibility, the person is held accountable for the success or failure of an obligation, not for their past behaviours per se. Under Feiring’s model [20], an individual who causally contributed to their condition should be given equal priority at the first instance of medical need. To have equal access to future healthcare, however, they would need to commit to a lifestyle contract and attend medical follow-ups, where a lifestyle contract is, broadly speaking, an agreement entered into by a patient to participate in specified health behaviours or make behavioural changes. Failure to agree to, or fulfil, the contract would result in lower priority access to public healthcare. This alternative approach to responsibility in healthcare may be a way forward in the ethical and political debate. We propose a modified version of Feiring’s prospective lifestyle contract model which has both pragmatic and normative benefits over the retrospective model, particularly in the context of the development of mobile health (mHealth) technology.

mHealth technology is quickly changing the landscape of healthcare. mHealth technology is defined by the World Health Organization (WHO) as “medical and public health practice supported by mobile devices, such as mobile phones, patient monitoring devices, personal digital assistants, and other wireless devices” [53]. In this paper, we focus on the specific subsection of mHealth technologies targeted at monitoring or improving health behaviours, including devices such as Fitbits or pedometers. In addition to providing personalised health information, technology may also provide a means to hold individuals accountable for their lifestyles. Health metrics and behaviours measurable by wearable mHealth devices have expanded to include transdermal blood alcohol levels [5], smoking hand gestures [15], and compliance with oral medications [3]. In the context of a growing burden of lifestyle-related diseases, the capacity to monitor key unhealthy behaviours has implications for the responsibility in healthcare debate. As a cautionary note, the mere existence of mHealth technologies does not necessarily mean they should be used. Some technologies may be judged as too risky or too difficult to regulate, and therefore limited in their practical applications in a healthcare setting.

The combined kindling of fiscal austerity, an individualistic, neoliberal culture extending into healthcare [7, 33], and a scarcity of resources may increase interest in rationing healthcare based on patient responsibility for ill health. This makes it crucial to reconsider the ethics of such policies, to propose models of responsibility that avoid disproportionately harsh and punitive treatment of individuals, and to consider how rapidly developing health technologies may alter how responsibility for lifestyle-related illnesses is attributed. Most critically, in democratic societies, the political feasibility of new health policies can depend on public attitudes, for a proposed framework of individual responsibility in health which has both theoretical and public support may be more readily adopted into practice. It is imperative, therefore, that we come to a greater understanding of what public attitudes relating to mHealth and responsibility in healthcare are.

Our research aims to achieve the following three goals: (1) to explore public attitudes towards different methods of incorporating responsibility into healthcare allocation; (2) to explore public willingness to use mHealth technology to help determine responsibility; and ultimately (3) to build on Feiring’s [20] lifestyle contract model and show that our empirically informed conceptual analysis supports a prospective framework of responsibility over a retrospective framework and that mHealth technology can serve as a useful adjunct in the prospective model.

## Methods

To meet our research objectives, we designed a survey that we administered to UK participants via Prolific, an online crowdsourcing platform that anonymously links researchers with potential study participants. Participants (n = 101) were UK citizens over the age of 18, gave informed consent, and were paid GBP2.40 for their time. The study was granted ethics approval by the University of Oxford’s Social Sciences & Humanities Interdivisional Research Ethics Committee.

The survey consisted of 31 items (see Online Resource 1), comprised primarily of case vignettes and 5-point Likert-type items. For all Likert-type items, ‘1’ represented complete disagreement and ‘5’ represented complete agreement. Five questions were duplicated with permission, from an (as yet) unpublished companion study investigating medical practitioner attitudes towards responsibility as a priority-setting criterion in healthcare. Of these items, one is duplicated from Bringedal and Feiring’s survey [11] of Norwegian physicians. Two Instructional Manipulation Checks (IMCs) were included to screen for inattentive responses. Of the total 101 participants, 81 participants passed both IMCs and were included in the data analysis. Statements presented in a matrix format were randomised to control for order effect, unless the statement was order dependent. All questions were compulsory, barring those which requested participant demographics. Items 1-3 were not included in the supplementary material (Online Resource 1) as they were preliminary questions regarding participant eligibility and consent.

Data analysis was conducted using IBM SPSS version 25 for Windows. Data obtained from Likert-type items was assumed to be normally distributed interval data, a decision which was made having considered the ongoing debate regarding the use of parametric tests on Likert-type data [10, 30, 49, 54]. Paired-sample t-tests or repeated measure ANOVA tests were used to assess differences across Likert-type items. McNemar–Bowker tests for asymmetry were used on categorical data obtained from case vignettes. The null hypothesis was rejected at *p* < 0.05.

Our survey investigated the following topics.

### General Attitudes Towards Individual Responsibility and Rationing Public Healthcare

5-point Likert-type items were used to assess the perceived acceptability of different justifications for rationing healthcare based on responsibility. We assessed whether participants believed people should be responsible for past behaviours on retributivist grounds (“just because that is what they deserve”) or consequentialist grounds (“because it will produce better outcomes for society”)(see item 5 of survey in Online Resource 1).

We also tested whether participants believed that there is a duty to oneself, one’s family, or one’s society to make healthy choices (see item 6 of survey in Online Resource 1).

### Prospective Versus Retrospective

Each of the first two case vignettes (see Table 1) (see questions 18 and 19 of survey in Online Resource 1) presented one patient prima facie responsible for their medical need and one who was not, both of whom required the same scarce treatment. The ‘responsible’ patient was willing to try to maximise medical benefit gained, unlike the ‘not responsible’ patient, who was unwilling to engage with an additional health-improving intervention. The participant was required to prioritise one of the two case patients or to grant them equal priority. Prioritising the ‘responsible’ patient was interpreted as indicating a preference to allocate resources based on a prospective framework of responsibility, while prioritising the ‘not responsible’ patient was interpreted as indicating a preference to allocate resources based on a retrospective framework of responsibility.

**Table 1:** Summaries of cases blood clots and hepatitis which assess participant preference for resource allocation based on prospective or retrospective responsibility

	Case blood clots	case hepatitis
Patient ‘responsible’ for illness (the ‘responsible’ patient)	Smoker but willing to take blood thinning medications	Contracted hepatitis due to needle sharing but willing to have medication compliance monitored
Patient not ‘responsible’ for illness (the ‘not responsible’ patient)	Genetic disorder increasing risk of blood clots but unwilling to take blood thinning medications	Contracted hepatitis through contaminated blood transfusion, but unwilling to have medication compliance monitored
Resource being allocated	Expensive treatment for damage due to chronic lung clots	Expensive course of curative therapy for hepatitis

### Do Intentions Matter?

A further two case vignettes measured whether participants valued the mere attempt to make healthy lifestyle choices or the ultimate outcome (Table 2). We compared a patient who had tried unsuccessfully to quit an unhealthy behaviour with a patient who had never attempted to quit in Case Alcohol Cessation, and with a patient who successfully quit in Case Smoking Cessation (see item 21 and 22 of survey provided in supplementary material).

**Table 2:** Summaries of cases alcohol cessation and smoking cessation which assess whether participants preferred to allocate resources based on attempt or outcome of lifestyle change

	Case alcohol cessation	Case smoking cessation
Patient X	Tried unsuccessfully to cease drinking	Tried unsuccessfully to cease smoking
Patient Y	Never attempted to cease drinking	Tried successfully to cease smoking
Resource being allocated	Liver transplant	Expensive cancer treatment

### Different Methods of Holding Patients Accountable Using Lifestyle Contracts

Concerns regarding the use of mHealth technology to hold people accountable for lifestyle choices were assessed, including concerns about personal and digital privacy, social equality, and the effect on the doctor–patient relationship. These were assessed in the context of both retrospective and prospective models of responsibility.

In response to a case vignette, participants indicated the perceived permissibility of several lifestyle contract monitoring methods, including no monitoring, traditional history taking, online self-reporting, and mobile health trackers.

## Results and Preliminary Discussion

101 respondents completed the survey. 81 respondents passed both attention checks and were included in the data analysis. As summarised in Table 3, 55.6% of participants were between the ages of 18–34. 69.1% were female and 54.4% identified with some degree of left-wing political leaning. Almost half (45.7%) had previously used or were currently using a wearable device or health app.

**Table 3:** Demographic information of participants

Demographic information	Category	% of total participants
Gender	Male	30.9
	Female	69.1
	Non-binary	0
	Prefer not to say	0
Age	18–24	23.5
	25–34	32.1
	35–44	21.0
	45–54	8.6
	55–64	9.9
	65+	4.9
	Prefer not to say	0
Highest education level	Primary school education	0
	Secondary school education	27.2
	Undergraduate tertiary education	48.1
	Postgraduate tertiary education	24.7
	Prefer not to say	0
Political orientation	Very left-wing/liberal	11.1
	Moderately left-wing/liberal	19.8
	Somewhat left-wing/liberal	23.5
	Neither left-wing/liberal nor right-wing/conservative	28.4
	Somewhat right-wing/conservative	7.4
	Moderately right-wing/conservative	6.2
	Very right-wing/conservative	0
	Prefer not to say	3.7
Digital tracker usage status	I currently use a wearable device/health app	30.9
	I have but no longer use a wearable device/health app	14.8
	I have never used a health app or wearable device	53.1
	Prefer not to say	1.2
Smoking status	Never smoked	53.1
	Ex-smoker	29.6
	Current smoker	17.3
	Prefer not to say	0
Body mass index	< 18.5	8.6
	18.5–25	33.3
	25–30	21.0
	> 30	11.1
	Don’t know/prefer not to say	25.9
Frequency of alcohol consumption over the last 12 months	Never	18.5
	Less than monthly	28.4
	Once or twice a month	22.2
	1–3 times weekly	23.5
	4–5 times weekly	3.7
	Daily	3.7
	Prefer not to say	0

### General Attitudes Towards Individual Responsibility and Rationing Public Healthcare

We investigated whether participants believed people have a duty to look after their own health, and if so, to whom this duty is owed. An overwhelming majority (93.8%) agreed either partly or completely that we owe ourselves such a duty, while 79.0% agreed either partly or completely that we owe our families the same. Interestingly, 64.2% agreed that we owe a duty to society to look after our own health. It seems that, even in a relatively individualistic society, there is nonetheless a sense of duty that extends beyond one’s immediate social circle. This may be particularly true in countries, like the UK, where healthcare is primarily accessed through the public system. With most participants believing in a duty to society regarding our own health, it may follow that failing this duty justifiably warrants consequences.

We also investigated general attitudes towards retributivist and consequentialist rationales for holding others responsible. The retributivist position, which holds that punishing wrong doers is of intrinsic moral worth, was represented by the statement “A person should be held responsible for their past choices just because that is what they deserve.” The consequentialist rationale for holding people responsible because it promotes utility was represented by the statement “A person should be held responsible for their past choices because it will produce better outcomes for society.” Our findings suggested that participants disagreed, either partly or completely, with holding people responsible for past behaviours both on retributivist (72.8%) and consequentialist (54.3%) grounds. The difference in approval levels between the retributivist and consequentialist positions was statistically significant [t(80) = − 6.40, *p* < 0.001], with the consequentialist position being the less objectionable of the two.

That people disagreed with holding others responsible is surprising and requires some explanation. In many domains of ordinary life, people often ascribe blame for perceived wrong-doing and hold the wrong-doer responsible, typically when the circumstances are such that the wrong-doer has a sufficient degree of intentional action, causal link to the given harm, and foresight that such a harm may arise [1]. The most likely explanation for our finding is that priming effects from the preceding question on healthcare may have inadvertently contextualised the statements, and that participants believed that while people ought to be held responsible in ordinary domains of life, the same does not apply in the special domain of healthcare. This is partially supported by our finding that in response to the 5-point Likert-type item “Healthcare priority should depend on the patient’s personal responsibility for the disease”, participants disagreed overall, albeit not strongly (M = 2.96). In the context of healthcare, ill health could itself be considered a natural punishment, or that the withholding of treatment is a disproportionately severe consequence for the relatively minor transgression of an unhealthy lifestyle.

### Prospective Versus Retrospective Responsibility

We found that participants were more favourable towards the notion of a prospective lifestyle contract than of retrospective responsibility. Of respondents, 65.4% agreed (M = 3.68) that priority access to healthcare should be lowered if multiple lifestyle contracts are broken, while 46.9% (M = 3.11) agreed with lowered priority access for a single broken lifestyle contract. In contrast, only 40.8% (M = 2.96) of participants agreed that healthcare priority should depend on whether the patient was personally responsible for the disease. These results suggest that while participants were overall ambivalent towards using retrospective responsibility for ill health as a resource allocation factor, lifestyle contracts based on prospective responsibility were better received.

We also tested participant preferences for either retrospective or prospective responsibility with two case scenarios (Table 1).

In Blood Clots, 50.6% of respondents prioritised the ‘responsible’ patient who contributed to her medical need but was willing to change her lifestyle to make the most of medical resources to receive expensive surgical treatment. Only 17.3% of respondents prioritised the ‘not responsible patient’ who was blameless for their medical need but would not change his lifestyle to help maximise the use of healthcare resources. The remaining 32.1% chose to toss a coin to allocate the treatment. This suggests that participants cared more about maximising benefits from a scarce resource than punishing patients who have contributed to their medical need. Our finding that people tend to place a high value on social utility when allocating healthcare resources is consistent with the current literature [4, 19, 21, 22, 26, 45, 47].

Although it was intended to reflect the same core moral principles, the case Hepatitis yielded dissimilar results. In comparison to the first case where 50.6% prioritised the ‘responsible’ patient willing to maximise responses, only 27.2% did so in the second case. 34.6% chose to prioritise the blameless ‘not responsible’ patient unwilling to maximise healthcare resources, while 38.3% chose to toss a coin. A McNemar–Bowker test revealed that participants were indeed responding asymmetrically across the Bloods Clots and Hepatitis cases [χ^2^(3, N = 81) = 18.57, *p* < 0.001].

We hypothesise that the reason for the discrepancy between the two response patterns is due to uncontrolled variables. In Hepatitis, Gabriel, the blameless ‘not responsible’ patient unwilling to maximise resources, requires medical care due to a contaminated blood transfusion. Unlike Gabriel’s homologue in Blood Clots, Gabriel has been harmed by the healthcare system and may have been perceived to have an additional claim to assistance. Furthermore, the ‘responsible’ patient in Hepatitis contracted the disease through the highly stigmatised behaviour of illicit intravenous drug use, potentially also skewing participants’ responses in Gabriel’s favour. It is notable that 27.2% of participants nonetheless opted to prioritise the ‘responsible’ patient who was more likely to produce the maximal benefits from the medication, despite their intravenous drug use.

Across these two cases, results suggest that participants do find patient willingness to take on responsibilities to produce the most expected good from a resource to be morally relevant. While this may be outweighed by other factors, such as being owed healthcare as a form of corrective justice, the concept of prospective responsibility appears to have some intuitive appeal. Notably, the cases presented demonstrate relatively clear causal relationships between behaviours and health outcomes. Public attitudes towards prospective responsibility in cases with complex, multifactorial aetiology, such as obesity, may be more difficult to interpret.

### Do Intentions Matter?

A further two cases examined how much moral weight participants placed on the intention and attempt to make positive lifestyle changes relative to the eventual outcome (see Table 2).

In Alcohol Cessation, participants were approximately evenly split between favouring the patient who tried to quit over the unsuccessful quitter and tossing a coin to allocate the liver (51.9% and 46.9% respectively).

In Smoking Cessation, a strong majority (70.4%) favoured the successful quitter, while 28.4% prioritised the patients equally.

Interestingly, of those who prioritised the well-intentioned but unsuccessful quitter in Alcohol Cessation, 78.6% prioritised the successful quitter in Smoking Cessation. Meanwhile, only 36.8% of those who chose the egalitarian ‘toss the coin’ option in Alcohol Cessation maintained this position into Smoking Cessation; of the remaining participants, all but 1.2% prioritised the successful quitter over the unsuccessful quitter.

Overall, these findings can be interpreted as participants placing some moral value in the mere attempt to change an unhealthy behaviour. A successful attempt, however, is perceived to have relatively more moral weight, as evidenced by the large percentage of participants who switched from prioritising the mere attempt to prioritising ultimate success. It is unclear why participants held this intuition. It may have been on a consequentialist rationale that the scarce resource would produce more benefit in an individual who has successfully given up a harmful behaviour. It may also be that participants attributed mental states or moral traits to patients based on whether they succeeded or failed a lifestyle change attempt [24, 34]. For example, participants may have unconsciously believed that the successful quitter tried more conscientiously or had more will power than the unsuccessful quitter, and therefore deserved higher priority for healthcare. Alternatively, participants may have believed that successful behaviour change is worth rewarding.

### Public Attitudes Towards Using Mobile Health Technology to Assess Responsibility in Healthcare

Participants held mixed opinions overall regarding the use of digital trackers instead of a discussion with a physician to assess lifestyle, with 44.5% agreeing and 40.8% disagreeing with such a practice. Due to the comparative strength of objections, however, the mean score (M = 2.90) for the Likert-type item indicated overall disagreement.

Predictably, participants had concerns about using mHealth technology to hold people accountable for their behaviours. We found that 59.3% of participants agreed either partly or completely that doing so would damage the doctor–patient relationship, while 65.4% agreed either partly or completely that people would problematically change their behaviours if they were being monitored. Interpretations of what constitutes problematic changes in behaviour might differ between participants, but might include making behavioural changes for undesirable reasons or taking advantage of flaws in the monitoring devices to misrepresent health behaviours.

While the difference did not reach standard statistical significance on this small sample [t(80) = − 1.96, *p* = 0.054], participants appeared slightly more likely to approve of using mHealth technology to monitor adherence to a lifestyle contract (M = 3.26) than of determining whether past behaviour contributed to illness (M = 3.01).

All ethical issues regarding mHealth included in the survey were deemed more problematic in a retrospective context than in a prospective context. Participants agreed that using data after the fact to assess past behaviours would pose unacceptable risks to data privacy (M = 3.60), more so than if it were used as part of a lifestyle contract (M = 3.30, t(80) = 2.79, *p* = 0.007). Participants also found mHealth technology to be fairer when assessing adherence with a lifestyle contract (M = 3.38) than when assessing past behaviour [M = 3.06, t(80) = − 2.93, *p* = 0.004].

Although not found to be statistically significant, participants were also more concerned about personal privacy in the retrospective framework (M = 3.6049) than in a prospective framework [M = 3.40, t(80) = 1.85, *p* = 0.068]. On a larger sample, this may have achieved statistical significance.

All four methods of monitoring adherence with a hypothetical lifestyle contract, which included no monitoring, traditional history taking with a physician, online self-report, and digital tracking, were perceived positively. Traditional history taking (M = 4.21) had the most support, and pairwise comparisons showed it was preferred over no monitoring (M = 3.28, MD = 0.93, *p* < 0.001), online self-report (M = 3.44, MD = 0.68, *p* < 0.001), and digital tracking (M = 3.35, MD = 0.86, *p* < 0.001). Means between the other methods were not significantly different. These results suggest that while participants prefer traditional methods of assessing compliance with prescribed lifestyle changes, digital methods would also be acceptable as part of the process. The survey did not elucidate participant attitudes towards the role which digital technologies might play in monitoring lifestyle changes, for example whether they ought to be used as a sole monitoring method or an adjunct to more traditional methods of healthcare provision.

Our survey findings should be considered exploratory; their generalisation is limited due to the skewed sample demographic, small sample size, and convenience sampling. Furthermore, it is prudent to note that our survey did not require participants to express the reasoning behind their intuitions and views, limiting hypotheses regarding the basis of people’s intuitions. Though the ensuing discussion will be guided by our findings, we acknowledge that more robust empirical evidence is required (Table 4).

**Table 4:** Summary of survey findings

Our key findings were	
1.	Participants believed in a duty to look after one’s health owed to oneself and, to lesser degrees, one’s family and society
2.	Violated lifestyle contracts were more acceptable than personal responsibility for disease as justifications for lowering an individual’s future healthcare priority, particularly if multiple contract violations occurred.
3.	Participants appeared to prefer holding patients prospectively responsible than retrospectively responsible, when doing so was a more efficient allocation of scarce resources. This is consistent with their preference for a consequentialist rather than retributivist reasoning for holding others responsible.
4.	Participant responses suggested that the mere attempt to make a healthy lifestyle change, even if unsuccessful, has some moral value. However, they preferred that scarce resources be allocated to individuals who had successfully made a change in comparison to individuals who had tried equally hard but were unsuccessful.
5.	Although participants had concerns about using mHealth technology to monitor lifestyles, it was significantly less objectionable in the context of a lifestyle contract than using the technology to reveal data about past lifestyle choices. Furthermore, while traditional history taking with a doctor was most preferred as a method of monitoring lifestyle contracts, a range of non-traditional methods, including digital tracking technology and online self-report, were also perceived to be acceptable methods of monitoring lifestyle contracts and may be useful as adjuncts

## Discussion

Based on key findings from our survey, it seems that the public prefers to allocate resources to individuals who would benefit the most, even if they are responsible for their illness.

These underlying beliefs are more consistent with a prospective, rather than a retrospective, model of responsibility. In our sample, people were attracted to lifestyle contracts and more willing to attribute responsibility on the basis of them, including when using mHealth technology. We will now propose a new model, then relate it to our empirical findings on relevant public intuitions in more detail.

### The Prospective Intention-Based Lifestyle Contract Model

With a growing burden of lifestyle-related diseases in a climate of fiscal austerity, there is the possibility of individual responsibility playing a greater role in public healthcare rationing. Philosophy does not exist in a vacuum. It may be that philosophical opposition to the responsibilisation in healthcare will be unable to counter political momentum. An alternative method of holding individuals responsible for health may provide a path that is politically palatable as well as philosophically less objectionable than traditional retrospective conceptualisations of responsibility in healthcare. It is not our intention to minimise the significance of social inequalities which contribute to poor health [35] or detract from the need for largescale public health initiatives; rather, we hope only to offer a harm reductionist approach to responsibilisation in healthcare.

At its core, Feiring’s [20] model proposes that an individual who contributed to their condition should be given equal priority at the first instance of lifestyle-related medical need. To have equal access to future healthcare, however, they must commit to a lifestyle contract supported by medical follow-ups to achieve the contracted goal to, for example, lose weight or quit smoking. Failure to agree to, or successfully fulfil, the contract would result in deprioritised public healthcare access. Building on Feiring’s [20] lifestyle contract model, we will now outline our version of the lifestyle contract model. It retains Feiring’s core idea but differs in several key aspects. Firstly, Feiring assesses fulfillment of a contract based on the ultimate outcome, while we require only that patients make a sincere attempt to make the contracted behavioural change. Secondly, we stress that the lifestyle contract should be reasonably achievable based on the patient’s social and environmental circumstances, and that appropriate support services be made available. Finally, our model shows how mHealth technology can be used as an adjunct to facilitate the assessment of whether a lifestyle contract has been fulfilled in addition to broadening the range of reasonably achievable lifestyle contracts.

**Fig. 1:**
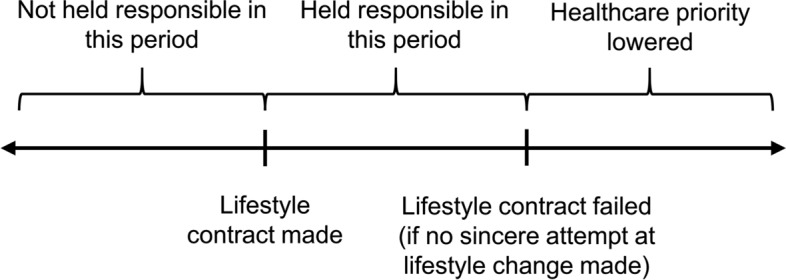
Schematic of lifestyle contract timeline

Under our proposed model, a patient whose lifestyle has contributed to their illness will have standard access at the first instance to scarce healthcare resources (Fig. 1). At this time, they will be given a lifestyle contract that commits them to certain conditions. If the contract is rejected or the requirements are not met, then their priority will be lowered for future instances of related healthcare need. The lifestyle contract should only contract behaviours that are likely to significantly improve the overall utility of the healthcare resource received. For example, the contract for a recipient of a liver transplant for alcohol-related liver disease should limit only risky behaviours that can reasonably be expected to damage the new liver, such as non-compliance with immunosuppressive medications or excessive alcohol intake.

Moreover, the lifestyle contract would assess the nature of the attempt to make a lifestyle change rather than the ultimate outcome. If the patient demonstrates a genuine attempt to change their behaviour, even if ultimately unsuccessful, then the patient is not deprioritised. For example, in the case of smoking, actively engaging with a formal cessation program may be sufficient to fulfil their contract. Bærøe and Cappelen [6] support a similar modification to Feiring’s lifestyle contract model, although they assess only whether a patient has attended relevant support services.

Requiring a sincere attempt at a lifestyle modification is less ethically problematic than Feiring’s model [20], as it represents a more realistic expectation of people. Furthermore, the contracted lifestyle modification should also be reasonably achievable for an individual’s socioeconomic circumstances which may impact a person’s ability to make the required change to varying degrees throughout a contract. For example, any resources or services required to fulfil the contract ought to be accessible and affordable, and any pre-existing social stressors which may limit the individual’s capacity to attempt to make difficult behavioural changes ought to be taken into account when drawing up the contract. These conditions are reminiscent of Savulescu’s and Davies’ concept of a Golden Opportunity [17, 44], which stipulates a set of conditions which need to apply to a health behaviour change in order for an individual to permissibly have health priority lowered based on responsibility if they choose to reject the change. These conditions include that the behavioural change must be realistically achievable for the individual and their circumstances, that the individual must be supported in making the behavioural change, and that there are clear consequences for failure to capitalise on the Golden Opportunity.

How, then, should a sincere attempt at a lifestyle change be assessed? An individual’s sincerity and motivation in pursuing lifestyle change are internal states inaccessible to direct measurement, leaving external behaviours or self-reflection as proxies. The lifestyle contract should be created in consultation with the patient based on the activities or measures they feel they would realistically be able to engage in. Possible measures of sincerity of an attempt include engagement with support services offered, for example, attending drug and alcohol support groups or participating in motivational interviewing sessions. Engagement with in-person professional services, however, is not always feasible due to geographical factors or other time commitments. Participation in online behavioural modification programs or tele-health services enabled by mHealth technology may be more suitable for some individuals. Another possible lifestyle contract assessment tool is an ongoing diary detailing the ways in which the patient attempted to make the relevant health behaviour change, in addition to circumstances or thought patterns which prevented them from doing so. Here, mHealth could serve as a useful adjunct in collecting data on individual behaviours, intentions, and attempted improvements. While a physical diary may be preferable for some patients, they have the limitations of being cumbersome and conspicuous to use. In contrast, mobile phone usage is often already part of a daily routine, and logging thoughts and events digitally would be less socially conspicuous. The individual could also set periodic reminders to complete the diary. Further adding to the benefit of mHealth enabled versions of the diary, data obtained from wearable devices regarding, for example, medication adherence or blood alcohol level, could automatically be integrated with a partner app, allowing the generation of digital graphs or trends which illustrate patterns in the individual’s behaviour. Problematic behavioural patterns could then be identified and any triggers or circumstances which lead to poor health choices could be mitigated. For example, a wearable device detecting medication adherence might find that over a period of several months, a patient’s adherence is worse after night shift. Based on the identification of this pattern, the patient could trial targeted strategies such as setting timed reminders.

Data from mHealth devices alone would not be grounds for failing a lifestyle contract, as it would be insufficient to assess the sincerity of the attempt at behavioural change, however it may be used to support an overall assessment of an individual’s behavioural change efforts. mHealth devices thus serve dual functions of supporting patients by providing information to troubleshoot unhealthy habits and improve health literacy, as well as providing objective data to support the assessment of whether a patient has fulfilled their lifestyle contract. The supportive function may also help dampen the potential negative impact on the individual’s relationship with their monitoring device.

### Empirically Aligned with Public Intuitions

In comparison to retrospective models of personal responsibility in healthcare, our proposed prospective model is better aligned with the public intuitions elicited in our exploratory survey. It balances efficient resource allocation with patient accountability for lifestyle-related illness, while improving the capacity for responsibility and providing a framework for individuals to take on greater agency in their health narrative [42].

Retrospective models of personal responsibility are usually based on desert-based claims, whereby if a patient foreseeably and avoidably caused their disease, then they have weaker claim to scarce public resources. Our results suggest, however, that people are more concerned with maximising the benefits derived from public resources.

A lifestyle contract represents an actionable duty to society to look after one’s health which, our findings suggest, is generally supported by the public. If that duty is unacceptably violated and scarce resources are underutilised by a patient, then consequences follow in the form of lowered healthcare priority.

Just as our study participants felt the attempt to make a healthy lifestyle change had some value, our model also allows that a sincere attempt, even if unsuccessful, to make the most of a scarce resource through behavioural change is sufficient to do due diligence to one’s duty to society.

Finally, mHealth technology acts as an efficient and publicly acceptable tool to transparently quantify adherence with lifestyle contracts.

### Theoretical Advantages

Our prospective lifestyle contract model also has theoretical advantages over retrospective models. Using Bærøe and Cappelen’s [6] excellent summary of the major criticisms of personal responsibility in healthcare as a framework, we will consider the same objections against using responsibility in healthcare: (1) the harshness objection; (2) the avoidability objection; (3) the intrusion objection; and (4) the objection of causality. Additionally, we will consider a fifth objection, the efficiency objection, which has also been used to argue against incorporating responsibility in healthcare. On each front, we will show why our prospective model of responsibility fares better than a retrospective model, particularly when the possible impacts of health monitoring technology are considered.

### The Harshness Objection and the Avoidability Objection

The related harshness and avoidability objections will be considered together.

At its core, the harshness objection argues that even if individuals are responsible for their illness, denying healthcare to them would be too harsh, punitive, and inhumane [14]. Lowering healthcare priority may result in inability to access treatment, longer periods of suffering, or even death [14]. Therefore, the policy should not be endorsed.

Meanwhile, the objection of avoidability argues that one can be responsible for an action only if it was possible to have done otherwise. If there is no genuine alternative available, then the agent cannot be held responsible.

Whether the prospective model is too harsh seems to depend in part on the nature of the lifestyle contract. If the contract is very difficult to uphold, then failing it should not be considered blameworthy, and deprioritised access to future healthcare would indeed be too harsh. If, however, the contract is reasonably achievable, then the harshness objection is weakened.

The avoidability objection now becomes relevant. Part of why lowering priority based on retrospective responsibility is harsh is because we know that the social determinants of health (SDH) are strong influences on an individual’s health behaviours and health outcomes [35]. The SDH are defined by WHO as “the conditions in which people are born, grow, live, work, and age” [56]. SDH include factors such as race, gender, disability, education, income, education, neighbourhood, and social support networks, many of which are beyond an individual’s control. Over the last two decades, evidence has emerged that SDH are closely linked with lifestyle choices, including poor diet, alcohol, smoking, and inactivity [36, 37, 48]. For example, fresh, healthy foods may prove unaffordable to those experiencing poverty, while poorer neigbourhoods have a higher density of fast food outlets and convenience stores [27], encouraging consumption of foods with poor nutritional value. Indeed, the circumstances of one’s birth and early development have a range of lasting effects on health [36], including the development of cognitive control that promotes behavioural regulation. With the complex and far-reaching effects of SDH in mind, there may be some individuals who, due to factors outside their control, would struggle to achieve a given lifestyle change in their respective circumstances. Some go as far as to argue that because external socioeconomic factors so strongly influence lifestyles and health outcomes, moral responsibility for lifestyle-related ill health cannot be attributed at all, as they may have been severely restricted in the range of choices genuinely available to them.

There are many factors which, if embedded in a lifestyle contract, would certainly render the contract not reasonably achievable and therefore too harsh a standard for allocating healthcare resources. To be considered reasonably achievable, the lifestyle contract would need to take an individual’s SDH and socioeconomic circumstances into account and attempt to recognise where barriers to success may lie. For example, it must be financially feasible relative to income, and take into account personal circumstances such as health literacy or geographic location. The individual should also be involved in determining what changes might be realistically achievable. The greater the hardship and barriers faced by the individual, the less that ought to be required in the contract, lest the profound impacts of the social determinants of health be underestimated. Input from social work and other psychosocial supports may aid in temporarily mitigating socioeconomic hardship, however efforts to actively overcome barriers to healthy lifestyle choices, such as poverty, would be limited by the scope of the healthcare system. These barriers would be better addressed by largescale public health efforts targeted at social inequalities and SDH.

Our proposed model acknowledges that behavioural changes are difficult, particularly in disadvantaged populations, and requires only an *attempt* to make a relevant behavioural change. If we assume there are far fewer people who could never make even a genuine *attempt* at behavioural change, then our model is less harsh as a standard to hold people to.

The core of the reasonably achievable lifestyle contract is to provide individuals with a genuine opportunity to try and make the most of the healthcare resources allocated to them. If an individual then fails to make a sincere attempt at a reasonably achievable contract while fully informed of the consequences, it does not obviously seem too harsh to deprioritise them. Even if we want to take into account the special importance of healthcare, perhaps by granting leniency in the number of reasonably achievable lifestyle contracts that one can fail before being deprioritised, an endless number of fresh starts is not owed by society.

Moreover, mHealth technology can increase the range of potential reasonably achievable lifestyle contracts. For example, behavioural modification programs delivered by mHealth devices or wearable lifestyle trackers is one method of overcoming geographical or time constraints, while emotional support might be made available through online social networks. Furthermore, mHealth devices can deliver health information at the individual’s own pace or in their preferred language.

Unless we are willing to discard intuitions about the existence of individual agency, there will be at least some circumstances where requiring a competent patient to make a genuine attempt at improving their lifestyle in a reasonably achievable way does not seem too harsh. This is provided that the social determinants of health mitigating the individual’s locus of control are taken into account, the consequences of failing the contract are proportionate, and there is genuine scarcity of the required resource. It is notable that some of the empirical findings from our survey were derived from zero-sum cases, where resources could only be allocated to one of two patients; healthcare resource allocation is rarely so simplistic. There are, however, some resources which are clearly either scarce, such as organ transplants, or extremely costly to the public healthcare system, such as novel curative treatments for hepatitis C [39]. While it may indeed be too harsh to require lifestyle contracts for any kind of treatment of lifestyle-related illness, it applies to a lesser degree when the required resource is scarce or significantly burdens the public system. Furthermore, we do not support complete withdrawal of publicly funded healthcare should a patient refuse or fail a proposed lifestyle contract, as this would be a disproportionate consequence. A plausible midground might be requiring the patient to contribute co-payments for future healthcare, relative to their ability to pay.

### The intrusion objection

The intrusion objection argues that determining patients’ responsibility for behaviours, past or future, would be too demeaning and intrusive. A great deal of personal information may be required to determine whether someone’s behaviour contributed to an illness, and may include sensitive information about sexual behaviour, recreational drug use, or mental illness. Requiring individuals to reveal such information, according to the intrusion objection, would be disrespectful and humiliating, and should thus preclude the use of responsibility as a healthcare allocation criteria [29].

Bærøe and Cappelen [6] have argued that the prospective model of responsibility can stand up to the intrusion objection, provided that a lifestyle contract was offered to anyone whose condition could be improved by a lifestyle change, thus avoiding the need for information about past behaviour. This reads as too demanding. Many, if not all, conditions would likely benefit from a healthier lifestyle, which may extend to choices such as hazardous occupations, hobbies, sleep patterns, and locations of residence. If everyone, even those with relatively healthy lifestyles, were required to enter a lifestyle contract for equal priority, the contracted changes could become supererogatory.

In the contract-based prospective model, the monitoring method of lifestyle contract adherence collects targeted information about the specific behaviour outlined in the contract, which may be facilitated by mHealth lifestyle trackers. For example, if limited alcohol consumption is part of a lifestyle contract following a liver transplant, then only blood alcohol levels should be measured. Collecting information on only the contracted behaviour limits the intrusiveness of the lifestyle contract model.

mHealth may also alter the strength of the intrusion objection in other ways. Some may find revealing embarrassing information to an electronic device less confronting. After all, an electronic device is not capable of moral judgements or disapproval. By removing interpersonal factors, using mHealth in the assessment of responsibility may lessen the degree of intrusion experienced by patients.

### The objection of causality

The objection of causality argues that (1) to be morally responsible for an event or state of affairs, it is necessary to be part of the relevant causal chain leading up to the given event or state of affairs and (2) we cannot determine whether voluntary behaviours caused any given disease state in many, if not all, cases. [25] This is particularly true of diseases with multifactorial aetiologies, where genetic predispositions, random gene mutations, and bad luck contribute in varying degrees.

Bærøe and Cappelen [6] argue that both the retrospective and prospective frameworks are vulnerable to the objection of causality, because both frameworks use the patient’s past behaviour to determine whether they are responsible for their medical need, either to lower priority or to determine whether a lifestyle contract should be offered.

Aside from diseases with only one aetiology, such as single gene disorders, it seems unlikely, at least in the foreseeable future, that we will be able to know what causally contributes to individual cases of illness and in what proportions. In a prospective model, a lower threshold of certainty could permissibly be applied to judgements of which patients were ‘responsible’ for their illness, because the consequences of doing so are less severe when offering a reasonably achievable lifestyle contract and only ensuring a sincere attempt than when directly deprioritising the patient. Although we cannot be certain of a patient’s causal role in illness in either a retrospective or prospective model, in a prospective model there is less need for absolute certainty.

### The objection of efficiency

According to the objection of efficiency, determining individual responsibility for ill health on a case-by-case basis would be extremely time-consuming, and would siphon healthcare resources from other areas of need [29]. It would, therefore, not be practically viable.

mHealth technology is particularly relevant here. Although not yet sufficiently sophisticated, innovations in mHealth technology will likely make it possible to monitor a wide range of physiological and physical parameters with minimal resources, such as blood alcohol level [5], calorie intake [3], smoking [15], and medication adherence [3].

Unless dramatically greater uptake of mHealth technology occurs, however, this is more advantageous for prospective models of responsibility than for retrospective models. Retrospective models would require the devices to have collected sufficient data and patient consent for data access. It is also plausible that people would avoid using mHealth devices to prevent future deprioritisation. These practical considerations limit the usage of mHealth technology to determine retrospective responsibility.

For a prospective model, however, mHealth technology may drastically reduce the resource intensity of assessing a sincere attempt to adhere to a lifestyle contract. It would likely decrease labour costs, as devices would take over some of the role of monitoring lifestyle contracts. Continuous data collection would allow general trends to be made apparent to health professionals without in-depth history taking and provide valuable information to help the individual and their supporting healthcare team to identify problematic patterns. mHealth may also enable mass distribution of behavioural change programs as part of a lifestyle contract, costing little to create and maintain.

### Limitations of the use of mHealth technology in lifestyle contracts

While the use of mHealth technology in the prospective model avoids some of the ethical concerns that arise in a retrospective model, it nonetheless poses possible risks. mHealth technology shares many of ethical difficulties beset by digital data as a whole, including risks to data privacy [12, 40, 51], murky data ownership laws [23], and poor oversight by regulative bodies [18, 32]. These issues also apply to existing health devices formally integrated into healthcare, such as continuous glucose monitors (CGMs) [12]. Ultimately, stronger, enforceable regulations on data management are needed to protect users [23].

Regarding the usage of mHealth technology in prospective lifestyle contracts, possible risks include the undermining of individual autonomy [41], intrusions into personal (as opposed to data) privacy [13], an antagonistic relationship between user and mHealth device, and inaccessibility to socially disadvantaged populations [2]. While an in-depth exploration of these issues is outside the scope of this paper, we will outline some general principles which aim to mitigate some of the ethical risks.

1. Voluntary—A lifestyle contract should not necessarily involve digital monitoring, nor should there be a penalty for withdrawing from a lifestyle contract’s digital monitoring component, particularly if the patient finds using digital devices stressful or harmful.
2. Minimising intrusion—The device should be limited to collecting data directly relevant to the lifestyle contract and be discussed with the patient prior to the contract’s creation. The device itself should be unobtrusive. Social functions, such as sharing with other app users, should be limited.
3. Accessible—The patient should bear minimal costs from using the device. Patients should be adequately supported and educated in the use of the devices. Any health apps involved in the contract should be available in a language comprehensible to the patient.
4. Accurate—The devices should be of high accuracy to avoid false representations of patient behaviours, which may be damaging to the doctor–patient relationship.

## Conclusion

Public healthcare systems are imperfect and rely on a limited pool of resources. Preventative healthcare and public health interventions are perhaps the most cost-effective measures to tackle the growing burden of lifestyle-related chronic disease [8, 38], and the effect of upstream social determinants of health on lifestyle-related chronic diseases cannot be underestimated. If, however, individual responsibility for illness becomes a more common rationing criterion, then our lifestyle contract model has several advantages over retrospective models.

The prospective intention-based lifestyle contract model is broadly supported by our key empirical findings. The model is concerned not with desert or punishment, but with the benefits that can be obtained from a limited pool of resources by a population in need. Our survey findings suggested that allocating resources to someone who was committed to making the best of a resource, even if they may have caused their illness, would be preferable to giving it to someone blameless but who would benefit less. Furthermore, the model recognises that successful behavioural change is challenging, particularly in the context of adverse socioeconomic circumstances, but that we can expect people to at least attempt to change their behaviours to maximise use of public goods. Finally, having found mHealth to be an acceptable tool, the lifestyle contract model also has the benefit of being able to utilise technology to support patients and provide an efficient means of holding them accountable.

## Data Availability

The datasets generated during and/or analysed during the current study will be available after publication in the Open Science Framework repository at https://osf.io/f6xnr/.

## Supporting Information

Below is the link to the electronic supplementary material. Supplementary material 1 (PDF 454 kb)
