# Supplementary material for: Prospective Intention-Based Lifestyle Contracts: mHealth Technology and Responsibility in Healthcare
Source: Health Care Anal. Author manuscript; Available in PMC 2021 Sep 1. (PMC8321967; doi:10.1007/s10728-020-00424-8)
Supplement: supplementary material [file EMS130973-supplement-Supplementary_Material.pdf]

Explanations of terms used in this survey:

For the purposes of this survey, if a patient enters into a lifestyle contract, it means they have formally agreed to make some kind of lifestyle change (eg. Quit smoking, increase physical activity levels, take medications as instructed). The lifestyle change usually relates to improving an existing medical condition or preventing other medical conditions from developing in the future.

* 4. To what extent do you agree/disagree with the following statements:

	Disagree completely	Disagree partly	Neutral	Agree partly	Agree completely
Healthcare priority should depend on the patient's personal responsibility for the disease.	◯	◯	◯	◯	◯
Access to expensive treatment should depend on the patient's personal responsibility for the disease.	◯	◯	◯	◯	◯
Access to scarce organ transplants should depend on the patient's personal responsibility for the disease.	◯	◯	◯	◯	◯
A patient who is responsible for the disease should pay extra to cover medical care.	◯	◯	◯	◯	◯
Lower priority should be allotted to patients who violate a contract of changes in lifestyle.	◯	◯	◯	◯	◯
Lower priority should be allotted to patients who violate a contract of changes in lifestyle multiple times.	◯	◯	◯	◯	◯
Whether a patient is considered responsible for their illness should be a decision made by an independent group of experts not directly involved with the patient's care, rather than their doctor.	◯	◯	◯	◯	◯

* 5. To what extent do you agree/disagree with the following statements:

	Disagree completely	Disagree partly	Neutral	Agree partly	Agree completely
A person should be held responsible for their past choices just because that is what they deserve.	○	○	○	○	○
A person should be held responsible for their past choices because it will produce better outcomes for society.	○	○	○	○	○

* 6. To what extent do you agree/disagree with the following statements:

	Disagree completely	Disagree partly	Neutral	Agree partly	Agree completely
People owe it to themselves to look after their own health.	◯	◯	◯	◯	◯
People owe it to their families to look after their own health.	◯	◯	◯	◯	◯
People owe it to society to look after their own health.	◯	◯	◯	◯	◯

* 7. For each of the following conditions/behaviours, do you think it should influence the individual's priority in healthcare? Please answer 'yes', 'no', or 'do not know' for each condition/behaviour.

	Yes	No	Do not know
Overweight/obesity	○	○	○
Smoking	○	○	○
Excessive alcohol consumption	○	○	○
Drug abuse / recreational drug use	○	○	○
Lack of physical exercise	○	○	○
High risk sports leading to injury/disease (eg. Skiing)	○	○	○
Poor quality nutrition	○	○	○
Unprotected sexual intercourse	○	○	○
Violation of contract of changed lifestyle	○	○	○
Not taking required regular medications	○	○	○
Unhealthily low body weight	○	○	○
Leave row blank if you are reading this	○	○	○
Combination of the above factors	○	○	○

Ashley, a long time smoker, has been diagnosed with lung cancer and requires an expensive treatment regimen.

* 8. Do you agree with this statement: Depending on how much she smoked, Ashley should receive lower priority for healthcare (eg. paying more for healthcare).

- ◯ Agree
- ◯ Disagree
- ◯ Not sure

Ashley, a long time smoker, has been diagnosed with lung cancer and requires an expensive treatment regimen.

* 9. How heavily should Ashley have smoked for her to justifiably receive lower priority for healthcare, for example through higher healthcare payments?
(Ie. if she has smoked more than X amount, she should be given lower priority.)

Move the slider to indicate your response.

1 cigarette(s)/week 140 cigarettes/week

Ben is on a wait list for publicly funded bariatric (weight loss) surgery. The recommended amount of exercise is at least 150 minutes of moderate intensity exercise per week.

* 10. Do you agree with the following statement: Depending on how little he exercised, Ben should receive lower priority for surgery.

- Agree
- Disagree
- Not sure

Ben is on a wait list for publicly funded bariatric (weight loss) surgery. The recommended amount of exercise is at least 150 minutes of moderate intensity exercise per week.

* 11. How little exercise should Ben have done for him to justifiably receive lower priority for the surgery, for example through longer wait times for the surgery? (Ie. if he has exercised less than X amount, he should be given lower priority.)

Move the slider to indicate your response.

0 minutes of exercise/week — 150 minutes of exercise/week

Charlie has had an accident after cliff diving, and requires time and resource intensive surgery.

* 12. Do you agree with the following statement: Depending on how much/often she cliff dived, Charlie should receive lower priority for healthcare (eg. paying more for healthcare).

- ◯ Agree
- ◯ Disagree
- ◯ Not sure

Charlie has had an accident after cliff diving, and requires time and resource intensive surgery.

* 13. How many times should Charlie have cliff dived previously for her to justifiably receive lower priority for healthcare (eg paying more for healthcare)?

(Ie. If she has cliff dived more than X number of times, she should be given lower priority.)

Move the slider to indicate your response.

0 previous cliff dives — 50 previous cliff dives

* 14. In what proportions do you think that nature (biology), nurture (culture), and/or free will influence human behaviour?

For each factor, please enter your answer as a percentage (but do not include a '%' sign).

The three answers must add up to 100. If any of your answers are 0, please enter '0' in the relevant text box.

Nature (biological factors such as genes)

Nurture (cultural/environmental factors)

Free will

Suppose data from digital trackers (eg. Fitbits, smartwatches) or health apps (eg. Apple Health, Google Fit) was used by health officials to hold people accountable for lifestyle choices and health behaviours. For example, if someone's doctor advises them to exercise three times a week, a digital tracker could confirm whether they are actually doing so.

* 15. To what extent do you agree/disagree with the following statements:

	Disagree completely	Disagree partly	Neutral	Agree partly	Agree completely
It would change the doctor-patient relationship in a negative way.	○	○	○	○	○
Lifestyle should be assessed using data from accurate continuous digital behaviour trackers instead of an in-depth discussion with a doctor.	○	○	○	○	○
It would problematically change how people behave if they knew their behaviour was being monitored and that they could be held accountable for it.	○	○	○	○	○
It would be broadly unethical to use digital trackers. Please leave this row blank if you are reading this.	○	○	○	○	○

Suppose data from digital trackers (eg. Fitbits, smartwatches) or health apps (eg. Apple Health, Google Fit) was used by health officials to assess whether a person’s past behaviour contributed to their illness. For example, if data from an app that tracked a person’s diet was used to assess how much the person's behaviour contributed to obesity.

* 16. To what extent do you agree/disagree with the following statements:

	Disagree completely	Disagree partly	Neutral	Agree partly	Agree completely
Data from a wearable device or health app, if available and accurate, should be used to determine if the person’s lifestyle contributed to their disease.	○	○	○	○	○
It would unjustifiably intrude upon personal privacy.	○	○	○	○	○
It would pose unacceptable risks to data privacy.	○	○	○	○	○
It would be a fair way of assessing patient responsibility for lifestyle related health problems.	○	○	○	○	○

Suppose data from digital trackers (eg. Fitbits, smartwatches) or health apps (eg. Apple Health, Google Fit) was used by health officials to keep track of whether a person was adhering to a lifestyle contract. For example, if data from a wearable device could track whether someone is still smoking after agreeing not to.

* 17. To what extent do you agree/disagree with the following statements:

	Disagree completely	Disagree partly	Neutral	Agree partly	Agree completely
Data from a wearable device, if accurate, should be used to monitor whether an individual is adhering to a lifestyle contract.	◯	◯	◯	◯	◯
It would unjustifiably intrude upon personal privacy.	◯	◯	◯	◯	◯
It would pose unacceptable risks to data privacy.	◯	◯	◯	◯	◯
It would be a fair way of assessing patient responsibility for lifestyle related health problems.	◯	◯	◯	◯	◯

* 18. Danny and Evelyn are both diagnosed with a blood clot in the lungs. Danny is found to have a genetic clotting disease, while Evelyn is a heavy smoker. Both these conditions greatly increase the risk of blood clots.

It is explained to both that they are likely to develop future clots, but they could take steps to avoid this happening. Danny (with the genetic clotting disease), however, refuses the recommended blood thinning medications. Evelyn (who smokes) is willing to try to quit smoking and take the recommended blood thinning medications.

In the future, both Danny and Evelyn require treatment for lung damage due to repeated clots. If all else, including medical need, is equal, who should have priority in receiving the surgery?

- ○ Danny should have priority for the surgery
- ○ Evelyn should have priority for the surgery
- ○ Toss a coin

* 19. Faye and Gabriel have both contracted a viral disease which damages the liver. Faye contracted the disease through sharing needles, whereas Gabriel contracted it through a contaminated blood transfusion.

The treatment is expensive and strict adherence is important for the drug to be effective. Faye (who shared needles) is willing to have her medication adherence tracked digitally, but Gabriel (who received a contaminated transfusion) is unwilling to have his medication adherence monitored in any way.

Only one treatment round is currently available. If all else, including medical need, is equal, who should receive the medication?

- ○ Faye should receive the medication
- ○ Gabriel should receive the medication
- ○ Toss a coin

* 20. Mr Green is a life-long smoker. He grew up on a farm and all his family smoked. He has end stage emphysema and requires a lung transplant to survive. He is currently smoking. He is 45 and in otherwise good health.

Mr Brown is a non-smoker, but has end stage emphysema from a genetic condition which damages the lungs. He is 40 but in poor health and had major heart surgery last year. You can refer only one patient for lung transplantation next week.

- ○ Refer Mr Green
- ○ Refer Mr Brown
- ○ Toss a coin
- ○ Do not know

* 21. Harper and Ingrid both have liver disease, following many years of alcohol abuse. Harper sought professional help for alcohol abuse but was ultimately unsuccessful in the attempt to quit, whereas Ingrid never sought help. Both now require a liver transplant. The medical need and likely outcome is the same for both. A liver becomes immediately available. Who should the liver be allocated to?

- ◯ Harper (who was unsuccessful at quitting) should receive the liver
- ◯ Ingrid (who never tried to quit) should receive the liver
- ◯ Toss a coin

* 22. Jamie and Kay are both smokers who required lung transplants. A requirement of the transplant was to quit smoking. After the transplant, Jamie and Kay attended the same quit program, and tried with equal efforts to give up smoking. Jamie ultimately succeeded in quitting, but Kay did not. In the future, who should receive priority for scarce healthcare resources? Assume that whether the individual smokes has no impact on the effectiveness of the future treatment.

- ◯ Jamie (who successfully quit) should have priority
- ◯ Kay (who tried but did not successfully quit) should have priority
- ◯ Toss a coin

Olivia has a disease of the joints which has not responded to conventional therapies. She is eligible for a publicly funded, expensive treatment, but she must not drink alcohol for the duration of the 8-week-long treatment course, as alcohol makes the medication much less effective. Olivia agrees to the lifestyle contract of not drinking alcohol while she is on the medication.

* 23. To what extent do you agree/disagree with the following statements:

	Disagree completely	Disagree partly	Neutral	Agree partly	Agree completely
Olivia should be trusted to keep her promise to not drink alcohol for 8 weeks and should not be monitored in this respect.	◯	◯	◯	◯	◯
Whether Olivia is fulfilling the lifestyle contract should be regularly be assessed by her treating doctor through face-to-face meetings.	◯	◯	◯	◯	◯
Whether Olivia is fulfilling the lifestyle contract should be monitored through self-reported information on an internet-based platform (eg. Health app, web page).	◯	◯	◯	◯	◯
Whether Olivia is fulfilling the lifestyle contract should be monitored through digital technology (eg. Wrist wearable that can detect blood alcohol level).	◯	◯	◯	◯	◯
If Olivia breaks the lifestyle contract and as a result receives little benefit from the expensive treatment, she should be allotted lower priority in the future for scarce healthcare resources.	◯	◯	◯	◯	◯

Demographics

We will now ask for some information about you that will help us understand your responses. This information is fully anonymous.

* 24. What is your gender?

- Male
- Female
- Non binary
- Prefer not to say

* 25. What is your age?

- Under 18
- 18-24
- 25-34
- 35-44
- 45-54
- 55-64
- 65+
- Prefer not to say

* 26. What is your highest level of education?

- Primary school education
- Seconday school education
- Undergraduate tertiary education
- Postgraduate tertiary education
- Prefer not to say

* 27. How politically left-wing/liberal or right-wing/conservative are you?

Very left-wing/liberal	Moderately left-wing/liberal	Somewhat left-wing/liberal	Neither left-wing/liberal nor right-wing/conservative	Somewhat right-wing/conservative	Moderately right-wing/conservative	Very right-wing/conservative	Prefer not to say
○	○	○	○	○	○	○	○

* 28. Do you currently, or have you ever used, a healthy lifestyle app or a health wearable device (eg fitbit, smartwatch)?

- I currently use a wearable device/health app
- I have but no longer use a wearable device/health app
- I have never used a health app or wearable device
- Prefer not to say

* 29. What is your smoking status?

- Never smoked
- Ex-smoker
- Current smoker
- Prefer not to say

* 30. What is your BMI?

- <18.5
- 18.5-25
- 25-30
- >30
- Don’t know/Prefer not to say

* 31. Over the last 12 months, which of the following options best reflects the frequency of your alcohol consumption?

- Never
- Less than monthly
- Once or twice a month
- 1-3 times weekly
- 4-5 times weekly
- Daily
- Prefer not to say
